# Bacterial diversity and community characteristics of the sinus and dental regions in adults with odontogenic sinusitis

**DOI:** 10.1186/s12866-023-02917-7

**Published:** 2023-07-29

**Authors:** Jianyou Wu, Ming Zheng, Yan Zhao, Weihong Yin, Yutong Sima, Jinming Zhao, Xiangdong Wang, Jiang Lin, Luo Zhang

**Affiliations:** 1 Department of Stomatology, Beijing TongRen Hospital, Capital Medical University, Beijing, China; 2 Department of Otolaryngology–Head and Neck Surgery, Beijing TongRen Hospital, Capital Medical University, Beijing, China; 3 Beijing Laboratory of Allergic Diseases and Beijing Key Laboratory of Nasal Diseases, Beijing Institute of Otolaryngology, Beijing, China; 4 Department of Allergy, Beijing TongRen Hospital, Capital Medical University, Beijing, China

**Keywords:** Odontogenic sinusitis, Sinus cavity, Oral cavity, 16S rRNA sequencing, Microbiome

## Abstract

**Background:**

The microbiome plays a crucial role in odontogenic sinusitis (OS); however, the bacterial characteristics of the sinuses and connected dental regions in OS are poorly understood. In this study, nasal secretion samples were collected from 41 OS patients and 20 simple nasal septum deviation patients, and oral mucosa samples from dental regions were collected from 28 OS patients and 22 impacted tooth extraction patients. DNA was extracted, and 16S rRNA sequencing was performed to explore the characteristics and structure of the microbiome in the sinuses and dental regions of OS patients.

**Results:**

The alpha diversity of the oral and nasal microbiomes in OS patients was higher than that in controls. Principal coordinate analysis (PCoA) showed that oral samples clustered separately from nasal samples, and the beta diversity of oral and nasal samples in OS patients was higher than that in controls. The dominant phylum was *Bacteroidetes* in OS patients and *Firmicutes* in controls in both the oral and nasal cavity. The dominant genera in the oral microbiome and nasal microbiome of OS patients were similar, including *Fusobacterium, Porphyromonas* and *Prevotella*. Co-occurrence network analysis showed decreased microbial connectivity in the oral mucosa and nasal secretion samples of OS patients.

**Conclusions:**

Odontogenic infection promotes structural and functional disorders of the nasal microbiome in OS. The interaction of dominant pathogens in the nasal and oral regions may promote the development of OS. Our study provides the microbiological aetiology of the nasal and connected dental regions in OS and is expected to provide novel insights into the diagnosis and therapeutic strategies for OS.

**Supplementary Information:**

The online version contains supplementary material available at 10.1186/s12866-023-02917-7.

## Introduction

As an inflammatory disease of the nasal and paranasal sinus mucosa, chronic rhinosinusitis (CRS) is considered a severe chronic respiratory disorder threatening human health [1, 2]. It poses considerable challenges to patients’ quality of life [3] and healthcare costs worldwide [4]. Odontogenic sinusitis (OS) is a common form of CRS caused by infection of the maxillary teeth close to the maxillary sinus. OS is frequently encountered in routine otolaryngology and dentistry, and more than 10% of maxillary sinusitis is due to odontogenic causes [5]. Although the occurrence of OS is associated with some factors and conditions, including maxillary tooth infection or trauma, tooth extraction, odontogenic disease of the maxillary bone, or endodontic root canal treatment for maxillary odontogenic disease, the exact pathogenesis of OS is not fully understood [6].

Management of OS requires dealing with not only dental problems but also sinus problems and often requires endoscopic sinus surgery (ESS) [7]. However, CRS caused by dental pathology is frequently ignored [8] and may result in medical and surgical failure [9]. OS has been associated with a larger microbial burden and more microbial diversity than simple CRS, and antimicrobial therapy should address this difference [10]. Oral infections spread rapidly through the maxillary sinus and can also lead to peri-orbital cellulitis, blindness, and even life-threatening cavernous sinus thrombosis [11–13]. Therefore, identifying and eliminating the source of infection in OS is necessary to prevent and control the persistence of symptoms [14, 15].

Antibiotics are the mainstay of medical treatment for OS, but the microbes linked to the occurrence of OS are often resistant to antibiotics. In clinical trials, the choice of appropriate therapeutic schedule depends on the discrimination of the characteristics of the microorganisms [16]. Therefore, it is very important to fully understand the bacterial characteristics of OS for the selection of antibiotics tailored to the specific microbiota.

The microbiome plays a crucial role in OS, and there are differences between the microbiome associated with OS and those of other types of rhinosinusitis [17]. The OS microbiome is generally polymicrobial, but anaerobic species, namely, bacteria in the oral cavity and upper respiratory tract, predominate [15]. The microbiome present in pathological dental problems may be critical in causing OS [16].

However, few studies have revealed the bacterial characteristics of the sinuses and connected dental regions in OS. Therefore, further microbiological studies are urgently needed to investigate and clarify the bacterial characteristics of the sinus cavities and connected dental regions of OS. We aimed to identify the microbiome present in oral pathological mucosa and nasal secretion samples of OS patients and controls by using 16S rRNA sequencing to determine the cause of odontogenic diseases. To our knowledge, this is the first study to demonstrate the microbiological profile of the sinus cavities and connected dental regions in OS patients.

## Materials and methods

### Subjects

From October 2020 to October 2021, 41 patients with OS, 20 individuals with simple nasal septum deviation, and 22 patients with mandibular impacted tooth extraction who visited the Capital Medical University Affiliated Beijing Tongren Hospital (Beijing, China) during the same period were enrolled in this study. In this cohort, samples from 41 OS patients were collected as the experimental subjects, including 41 nasal secretions (experimental group nasal secretions, EGNS) and 28 oral mucosa (experimental group oral mucosa, EGOM). Nasal secretions (control group nasal secretions, CGNS) from 20 patients with simple nasal septum deviation and oral mucosa (control group oral mucosa, CGOM) from 22 patients with mandibular impacted tooth extraction were collected as controls. Participants were excluded if they were taking antibiotics or probiotics in the past month. Patients with simple nasal septum deviation were excluded from sinusitis by CT and nasal endoscopy, and there were no clinical symptoms of sinusitis.

### Sample collection

**Fig. 1:**
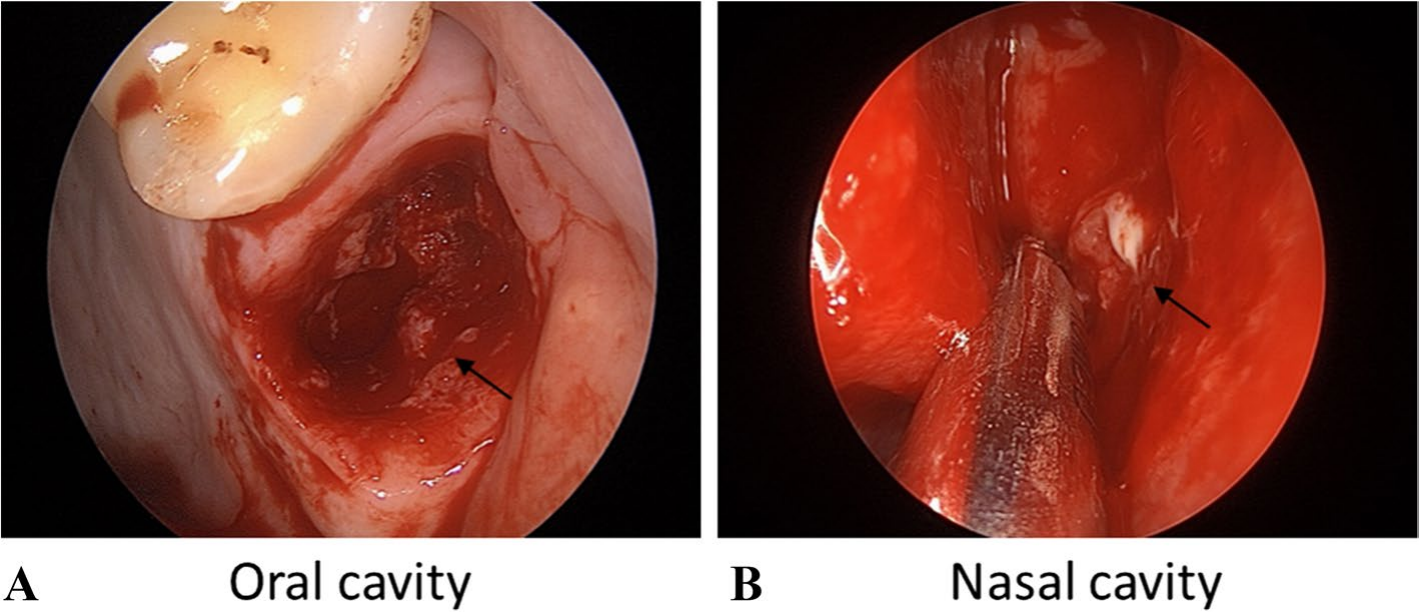
Sampling location. **A** Oral cavity extraction socket. **B** Nasal secretion. (The arrow points to the sampling location)

ESS and extraction of affected teeth were performed simultaneously, and oral pathological mucosa and nasal secretion samples were extracted in OS patients. Diseased granulation tissues or infected gingival tissues were scraped away at the depth of the extraction pit to collect oral pathological mucosa samples for the detection of dental bacteria (Fig. 1A). During ESS, the maxillary sinus ostium was opened, and sterile gauze was immersed into the maxillary sinus cavity to collect nasal secretions (Fig. 1B). When collecting nasal secretions from patients with deviated nasal septa, secretions from the middle meatus were thoroughly dipped in a sterile cotton swab and placed into a frozen tube containing DNA protection solution. For oral samples from patients with impacted teeth extraction, excess gum tissue was removed during the extraction and placed in a frozen tube. The samples were collected in sterile tubes and immediately stored at -80 °C for further processing.

### DNA extraction and 16S rRNA sequencing

Using the PowerSoil DNA Isolation Kit (MoBio Laboratories, Carlsbad, CA), DNA was extracted from mucosal and secretion samples according to the instructions. Genomic DNA purity and quality were measured on 0.8% agarose gels. The V3-4 hypervariable region of the bacterial 16S rRNA gene was amplified with the primers 338F (ACTCCTACGGGAGGCAGCAG) and 806R (GGACTACHVGGGTWTCTAAT) [18]. For each sample, a 10-digit barcode sequence was added to the 5’ end of the forward and reverse primers (provided by Allwegene Company, Beijing). Then, Mastercycler Gradient (Eppendorf, Germany) was used for PCR with 5 µl of DNA (total template volume was 30 ng). The cycling parameters were set at 95 °C for 5 min, followed by 28 cycles of 95 °C for 45 s, 55 °C for 50 s and 72 °C for 45 s, and finally extended to 72 °C for 10 min. To mitigate PCR bias at the reaction level, three PCR products from each sample were combined. The PCR products were purified using a QIAquick Gel Extraction Kit (QIAGEN, Germany), quantified using real-time PCR, and sequenced at the Allwegene Company (Beijing). Deep sequencing was performed on a MiSeq platform. Images were analysed, bases were called, and errors were estimated using Illumina Analysis Pipeline Version 2.6.

### Sequence analysis

The Quantitative Insights into Microbial Ecology (QIIME) pipeline was used to trim the raw sequences [19]. Briefly, the original sequences matching the bar codes were identified as valid sequences and assigned to their respective samples, and their primers and bar codes were pruned for further quality control. The filtering criteria for low-quality sequences were sequences of length 6. The remaining high-quality sequences were clustered into operational taxonomic units (OTUs) under 97% sequence consistency. OTUs are artificial markers for a taxon (family and genus, etc.) that can be used to classify identified sequences. Reads less than 150 bp in length, containing an ambiguous base, or containing an isoform greater than 8 bp were removed, and chimeric sequences were identified and removed using the UCHIME tool of the mothur software package (v.1.31.2) [20]. All clipped sequences were normalized to the same sequence depth using mothur.

### Statistical analysis

According to the OTU clustering results, rarefaction analysis was performed by mothur. The R language tool was used to make dilution curve rarefaction curves. R language tools for statistics and Venn plots were also used [21]. QIIME V. 1.8.0 was used to measure alpha diversity using the Shannon index and beta diversity using unweighted UniFrac and Bray‒Curtis distances [22]. To estimate the beta diversity, PCoA was performed using the R package vegan. Between-group variances was evaluated using analysis of similarities (ANOSIM). RDP Classifier, BLAST, and UCLUST consensus taxonomy assigner were used for comparative analysis of representative OTU sequences, and the species information for the OTU community was annotated at each level (phylum, class, order, family, genus). Differences in taxonomic composition were assessed using the Kruskal–Wallis or Wilcoxon rank-sum test.

The linear discriminant analysis effect size (LEfSe) method [23] was used to compare the bacterial community structures between the samples. ANOVA was used to detect species with significant differences in abundance between different groups, and the threshold was set at 0.05. Linear discriminant analysis (LDA) was used to reduce and evaluate the impact of species with significant differences (LDA score), and the threshold was set at 3 (*P* < 0.05, LDA > 3). To examine the cooccurrence or mutual exclusion of the top 20 genera, a correlation network was generated using mothur software, and calculated C-scores and Spearman's rank correlations were used to analyse cooccurrences among genera. Only correlations with a significance of *P* < 0.05 and a correlation of *r* > 0.6 were included in the network. Cytoscape software version 3.8.2 was used to visualize and edit the network.

## Results

### Demographic data

In this cohort, samples from 41 patients with odontogenic sinusitis (OS) were collected as the experimental group, including 41 nasal secretions (EGNS) and 28 oral mucosa samples (EGOM). For the control group, nasal secretions (CGNS) from 20 patients with simple nasal septum deviation and oral mucosa samples (CGOM) from 22 patients with mandibular impacted tooth extraction were collected. Specific demographic information is shown in Table 1.

**Table 1:** Demographic data for EGNS, EGOM, CGNS, and CGOM

Demographic data	EGNS (*n* = 41)	EGOM (*n* = 28)	CGNS (*n* = 20)	CGOM (*n* = 22)	***P* value**
Age (mean ± SD years)	46.73 ± 13.76	44.36 ± 13.31	34.65 ± 9.28	29.73 ± 7.67	< 0.0001
Male sex (n)	23	11	12	4	0.0140
Cause of disease	OS	OS	Nasal septum deviation	Impacted teeth	
Smoking	13	6	3	2	0.1705
Asthma	0	0	0	0	NA
Diabetes	6	2	1	0	0.2229

*EGNS* Experimental group nasal secretions, *CGNS* Control group nasal secretions, *EGOM* Experimental group oral mucosa, *CGOM* Control group oral mucosa

### Valid sequencing data

**Fig. 2:**
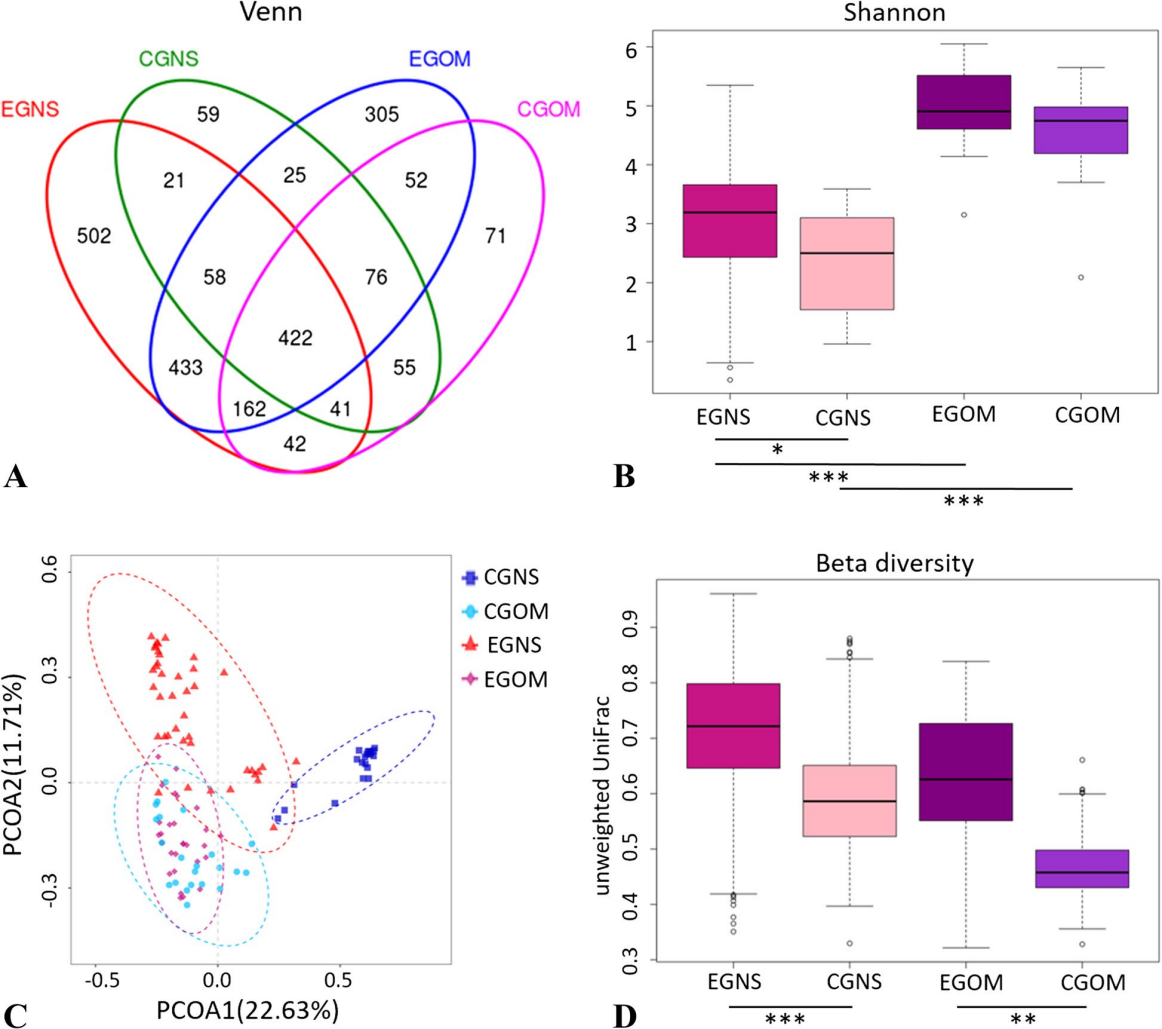
Venn diagram and diversities of the microbiota. **A** Venn diagram showing shared and unique operational taxonomic units (OTUs) at 97% identity among the four groups. **B** Alpha diversity measurements using the Shannon diversity index in each group at 97% identity. The data were tested by Tukey’s test. **C** Principal coordinate analysis (PCoA) based on the OTU level. **D** Unweighted UniFrac distance analysis of the microbiome in each group. Data were tested by ANOSIM. * *P* < 0.05, ** *P* < 0.01, *** *P* < 0.001. EGNS: experimental group nasal secretions; CGNS: control group nasal secretions; EGOM: experimental group oral mucosa; CGOM: control group oral mucosa

In total, 50 oral pathological mucosa and 61 nasal secretion samples were collected. All the rarefaction curves (Figure S1) reached saturation at approximately 25,000 sequences per sample, suggesting that the sampling was comprehensive and that the sequencing depth was sufficient to reflect the vast majority of microbial information. A total of 2324 operational taxonomic units (OTUs) were detected in all samples (Fig. 2A): 1742 were detected in the oral mucosa samples, accounting for 74.96% of all the OTUs; 1896 OTUs were found in the nasal secretion samples, accounting for the largest proportion (81.58%) of the total. Region-specific OTUs were also demonstrated, including 428 (18.42%) in the oral mucosa samples and 582 (25.04%) in the nasal secretion samples. In oral-specific OTUs, 502 were in EGNS, while only 59 were in CGNS. In nasal cavity-specific OTUs, 305 were in EGOM, while only 71 were in CGOM. Consequently, OTU counts of the nasal microbiome were more abundant; meanwhile, the numbers of region-specific OTUs in both pathological oral and nasal cavities of OS patients were higher than those of the control group, which might be associated with regional bacterial infections.

### Bacterial diversity in oral and nasal samples

To assess the overall composition richness and structural characteristics of the oral and nasal regions in OS patients, we analysed the alpha and beta diversity of the microbiota. Alpha diversity measurements using the Shannon diversity index (Fig. 2B) indicated a significant increase in the microbial diversity of oral mucosa samples (Tukey test, *P* < 0.001). Comparing the alpha diversity of nasal secretions, we found that the microbial diversity of OS patients was significantly higher than that of controls (*P* < 0.05). Similarly, oral microbial diversity was higher in OS patients, although there was no significant difference. To further understand bacterial community structures among oral and nasal regions, beta diversity was assessed by PCoA. The PCoA plot (Fig. 2C) showed that the microbiome of the oral mucosa samples clustered separately from that of the nasal secretion samples (ANOSIM, *P* < 0.001). Beta diversity analysed by unweighted UniFrac distance of oral mucosa (ANOSIM, *P* < 0.01) and nasal secretion samples (ANOSIM, *P* < 0.001) in OS patients was also higher than that in controls (Fig. 2D). These results indicated that the oral and nasal microbiomes significantly differed from each other. Moreover, there were differences in the distribution of the oral or nasal microbiome between OS patients and controls.

### Comparison of bacterial abundance in the oral and nasal samples

**Fig. 3:**
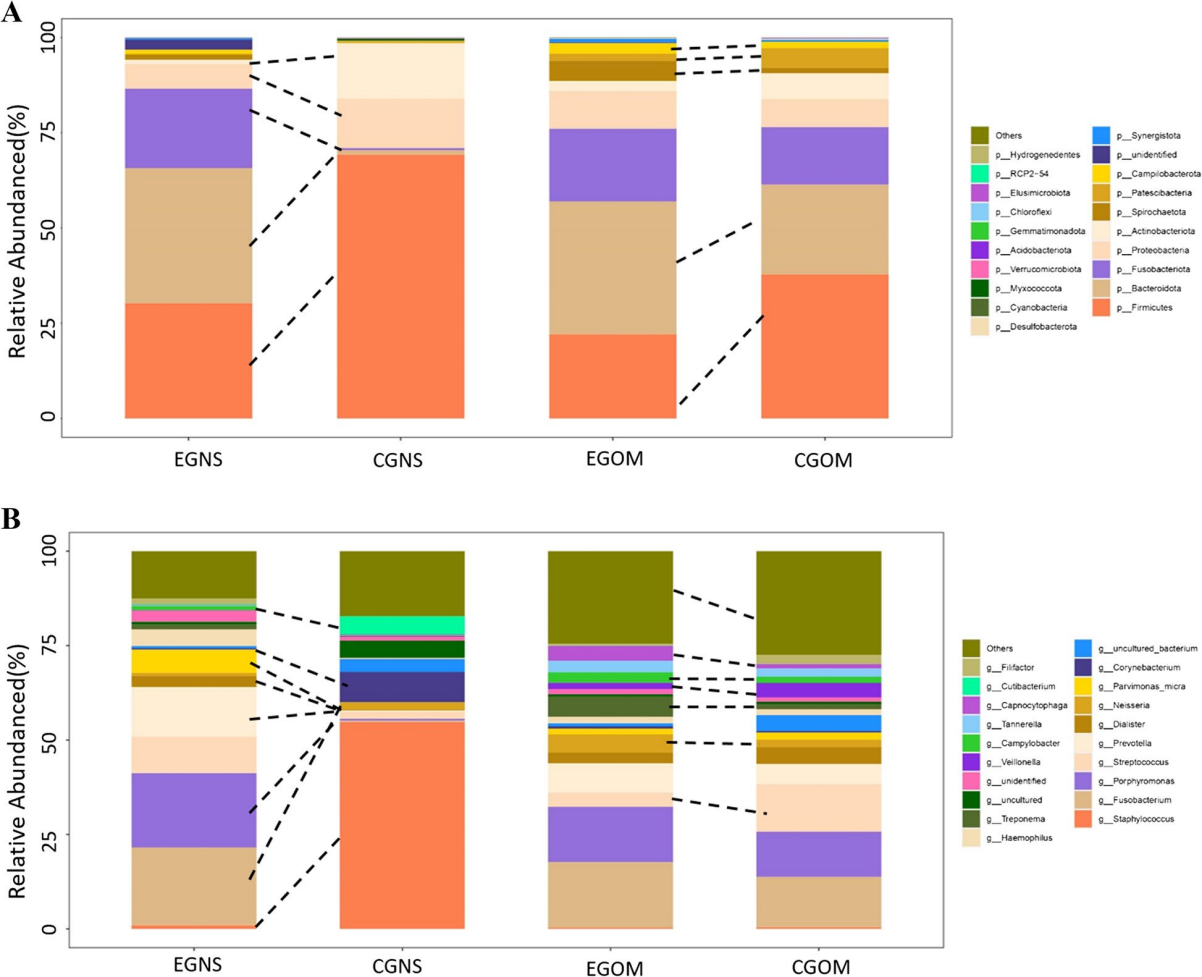
Histogram of bacterial composition analysis. **A** Community structure of the microbiome at the phylum level of different groups. **B** Community structure at the genus level (The abscissa represents the group, and the ordinate represents the relative abundance of bacteria in the group. The data showed a relative abundance of bacteria of more than 1%, and the dashed lines showed bacteria with significant changes.). EGNS: experimental group nasal secretions; CGNS: control group nasal secretions; EGOM: experimental group oral mucosa; CGOM: control group oral mucosa

To assess bacterial abundance, we subsequently performed a taxonomic analysis of the nasal and oral microbiomes. The distribution of bacteria was characterized by relative taxonomic abundance. A total of 36 phyla, 88 classes, 182 orders, 308 families, and 565 genera were identified in the samples. First, to determine if and how the microbial compositions varied between oral mucosa and nasal secretion samples, we calculated the relative abundances of the phyla in these regions. The taxon compositions of the microbial communities with a relative abundance of more than 1% according to the tested sample groupings are provided (Fig. 3A). The dominant phyla in EGNS mainly comprised *Bacteroidota*, *Firmicutes*, *Fusobacteriota*, and *Proteobacteria*, while *Firmicutes*, *Actinobacteriota* and *Proteobacteria* were dominant in CGNS samples. The proportions of *Actinobacteriota* (*P* < 0.05), *Firmicutes* and *Proteobacteria* (*P* < 0.05) were more abundant in CGNS, while the proportions of *Bacteroidota* (*P* < 0.05) and *Fusobacteriota* were more abundant in EGNS. Additionally, *Bacteroidota*, *Firmicutes*, *Fusobacteriota* and *Proteobacteria* were the dominant phyla in EGOM and CGOM. The proportions of *Firmicutes* and *Patescibacteria* were more abundant in CGOM, while the proportions of *Bacteroidota*, *Spirochaetota* and *Campilobacterota* were more abundant in EGOM.

The genera in the samples were sorted from most to least abundant (Fig. 3B). The 5 most abundant genera were *Fusobacterium*, *Porphyromonas*, *Prevotella*, *Streptococcus* and *Parvimonas_micra* in EGNS and *Staphylococcus*, *Corynebacterium*, *Cutibacterium*, *Peptoniphilus_sp._EL1*, *and Ralstonia* in CGNS. The 5 most abundant genera were *Fusobacterium*, *Porphyromonas*, *Prevotella*, *Treponema* and *Neisseria* in EGOM and *Fusobacterium*, *Streptococcus*, *Porphyromonas*, *Prevotella* and *Dialister* in CGOM. Additionally, we also analysed the differential genera with relative abundances in the top 20 in different regions of OS patients and controls. *Staphylococcus*, *Corynebacterium*, *Cutibacterium* and *Neisseria* were less abundant (*P* < 0.05), while *Fusobacterium*, *Porphyromonas*, *Prevotella*, *Parvimonas_micra* and *Dialister* were more abundant (*P* < 0.05) in EGNS than in CGNS. *Streptococcus*, *Filifactor* and *Veillonella* were less abundant (*P* < 0.05), while *Treponema*, *Neisseria*, *Capnocytophaga* and *Campylobacter* were more abundant (*P* < 0.05) in EGOM than in CGOM.

### High-dimensional biomarkers in different regions of OS

**Fig. 4:**
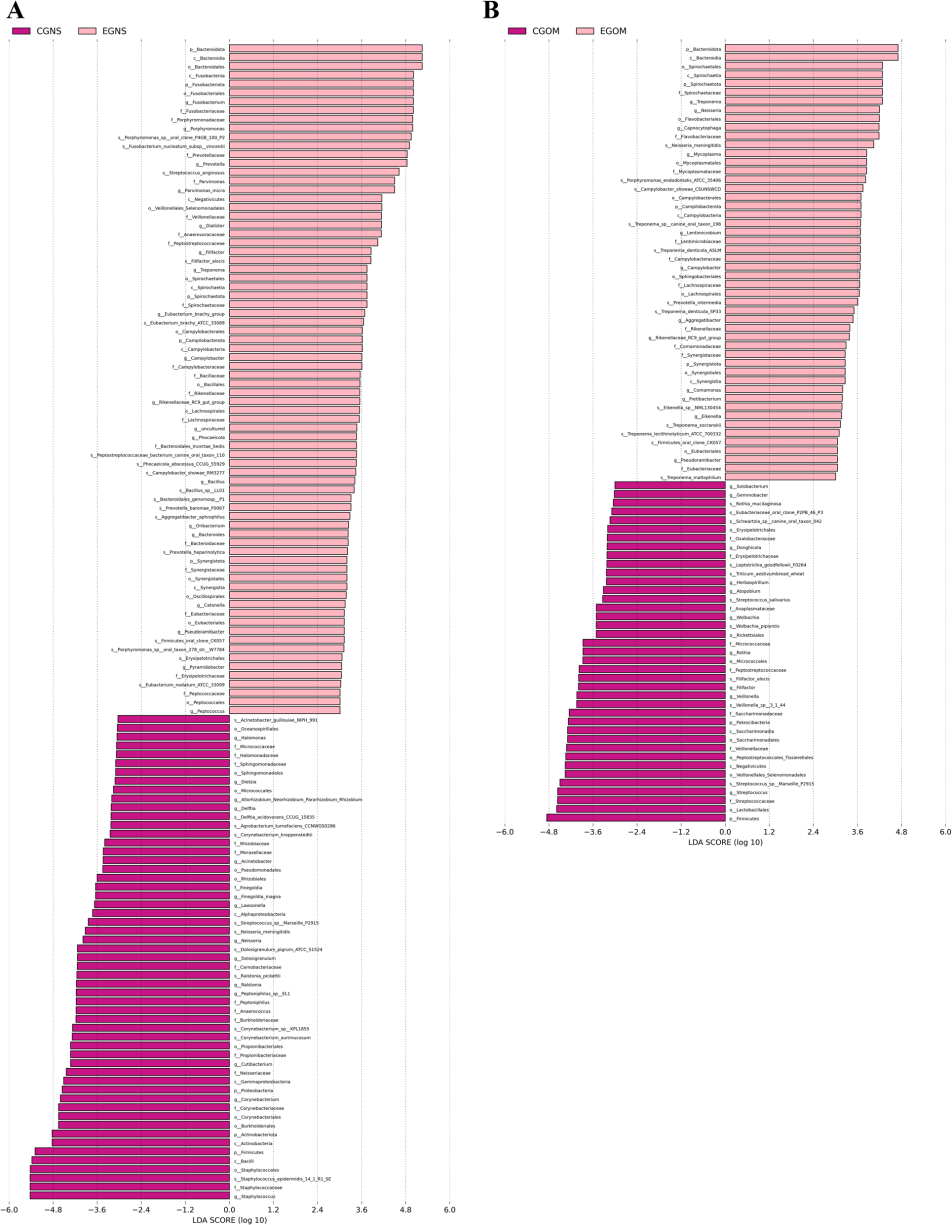
Linear discriminant analysis (LDA) score histogram for differential OTUs of nasal secretions (**A**) and oral mucosa (**B**) between OS and control. LDA scores were calculated among classes (Kruskal–Wallis test) and between subclasses (Wilcoxon’s test), and significant differences (*P* < 0.05) were produced. Differential taxa were identified according to a statistical significance level of 0.05 and an LDA threshold score of > 3 (g: genus, f: family, o: order, c: class, p: phylum). EGNS: experimental group nasal secretions; CGNS: control group nasal secretions; EGOM: experimental group oral mucosa; CGOM: control group oral mucosa

**Fig. 5:**
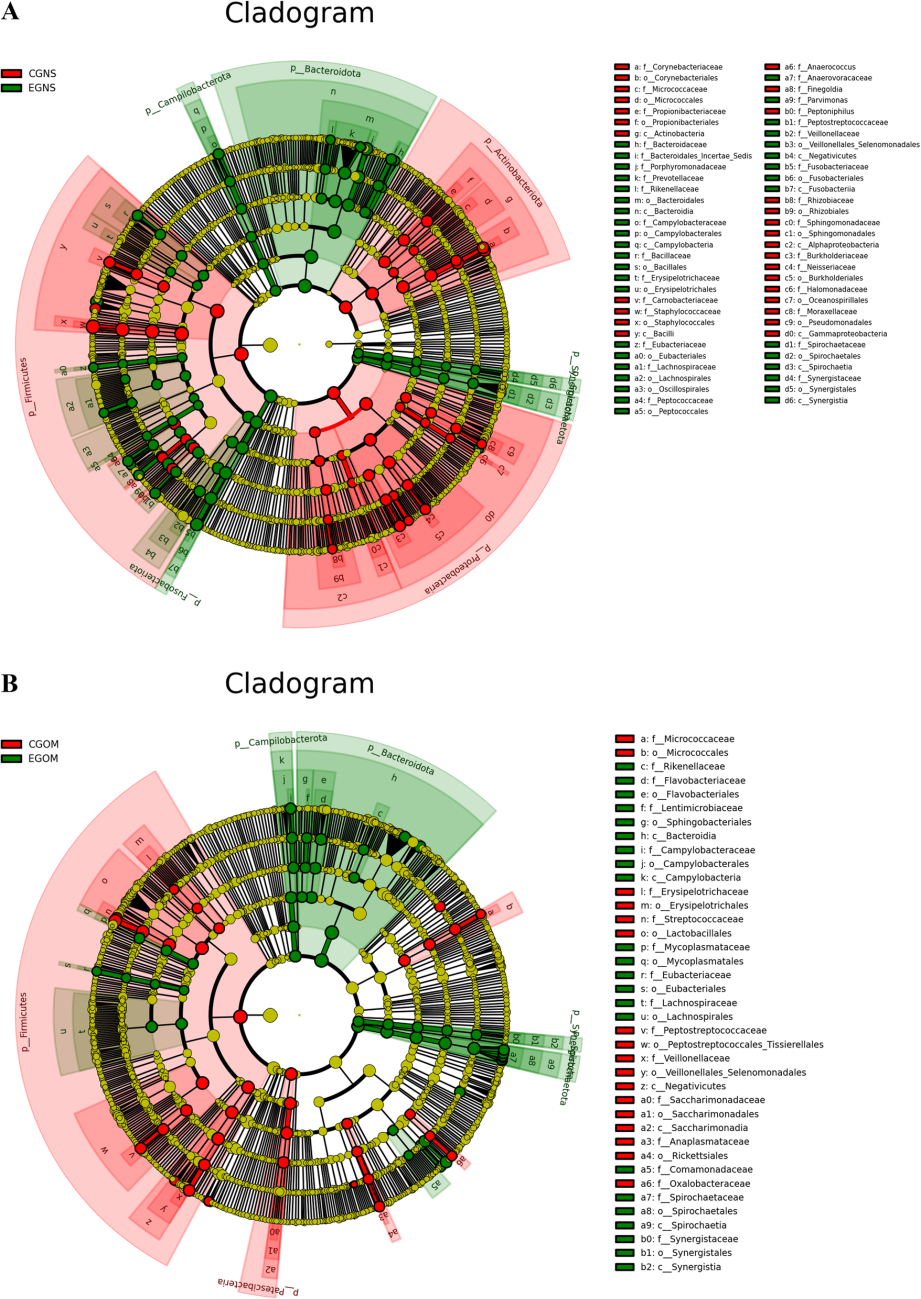
Cladogram for differential taxa of nasal secretions (**A**) and oral mucosa (**B**). Differences are represented in the colour of the most abundant taxa. **A** Red indicates nasal secretion in control, green indicates nasal secretion in OS patients; **B** Red indicates oral mucosa in control, green indicates oral mucosa in OS patients.) EGNS: experimental group nasal secretions; CGNS: control group nasal secretions; EGOM: experimental group oral mucosa; CGOM: control group oral mucosa

To further clarify the differences in oral or nasal regions between OS patients and controls, LEfSe was used to detect high-dimensional biomarkers to identify bacterial taxa. These differentially abundant taxa could be considered potential biomarkers (LDA Score > 3, *P* < 0.05) (Fig. 4). Pathology-specific biomarkers, especially biomarkers from oral mucosa and nasal secretions, could be used for OS prediction with high fidelity. The most significantly increased bacteria in nasal secretion samples were *Bacteroidota* and *Fusobacteriota*, while the most significantly decreased bacteria were *Staphylococcus* and *Bacilli* (Fig. 4A). The most significantly increased bacteria were *Bacteroidota*, *Bacteroidia*, and different taxa of *Spirochaetota*, while the most significantly decreased bacteria were *Streptococcaceae*, *Lactobacillales*, and *Firmicutes* in oral mucosa samples (Fig. 4B). To further analyse the detailed information on the differential bacteria, we employed cladograms to obtain a more comprehensive view of the bacterial taxonomy and spatial distribution of bacteria (Fig. 5). Among the differentially increased bacteria in nasal secretion samples, most were classified as *Bacteroidota*, followed by *Campilobacterota*, *Synergistia*, *Spirochaetota*, and *Fusobacteriota*; among the differentially decreased bacteria, most were classified as *Firmicutes* and *Proteobacteria*, followed by *Actinobacteriota* (Fig. 5A). In oral mucosa samples, most of the differentially increased bacteria were classified as *Bacteroidota* and *Campilobacterota*, followed by *Spirochaete* and *Synergistales*; most of the differentially decreased bacteria were classified as *Firmicutes*, and only a few were classified as *Patescibacteria* (Fig. 5B). These results suggest that *Bacteroidota, Fusobacteriota* and *Spirochaetota* may serve as significant markers of OS.

### Co-occurrence network analysis between oral and nasal samples of OS patients

**Fig. 6:**
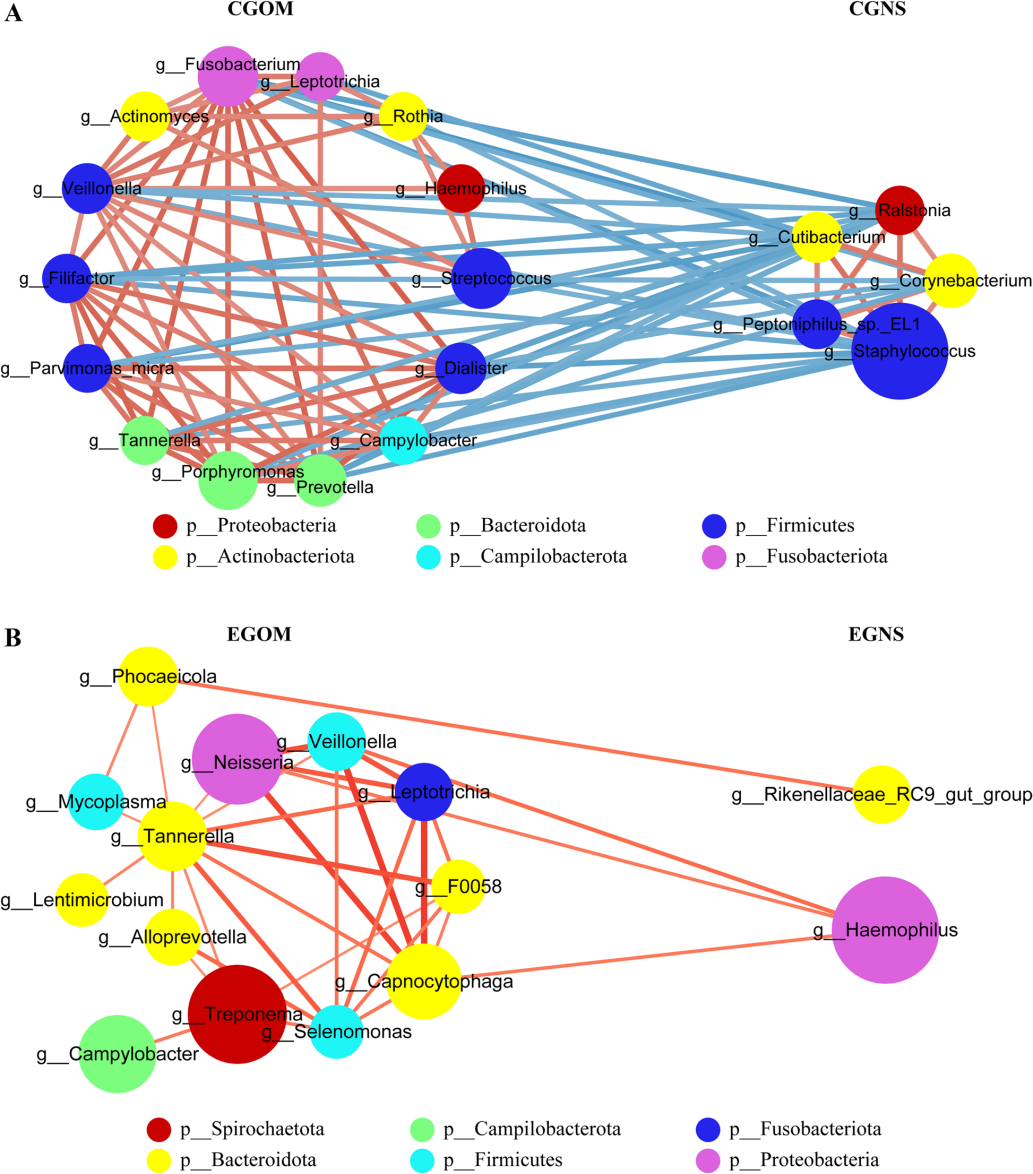
Network correlation diagram based on co-occurrence genera showing associations in nasal secretions and oral mucosa samples of control (**A**) or OS patients (**B**). The size of the point represents the abundance, and the thickness of the line represents the degree of correlation. Dots of the same colour represent the same phylum to which they belong, red for positive correlation and blue for negative correlation. EGNS: experimental group nasal secretions; CGNS: control group nasal secretions; EGOM: experimental group oral mucosa; CGOM: control group oral mucosa

To further study the interaction of bacteria between oral and nasal samples of OS patients, Spearman's correlation coefficient of the top 20 co-occurring genera was calculated in the two groups. Co-occurrence network diagrams representing strong (*r* > 0.6) and significant (*P* < 0.05) correlations were drawn using Cytoscape software. In the control group, a network of these genera in oral mucosa and nasal secretion samples was plotted with 19 nodes displaying associations based on the correlation analysis and connections (94 lines) (Fig. 6A). *Fusobacterium* (15 lines), *Veillonella* (14 lines), *Cutibacterium* (14 lines), and *Staphylococcus* (14 lines) were the main core nodes that closely interacted with other genera. Interestingly, decreased microbial cooccurrence network connectivity was observed in oral mucosa and nasal secretion samples of OS patients (Fig. 6B). The network of co-occurring genera in the oral mucosa was plotted with 15 nodes displaying associations based on the correlation analysis and connections (33 lines) in the network. *Tannerella* (11 lines) was the main core node that interacted closely with other genera. Most correlations were negative for oral and nasal samples within the control cluster but were positive between genera of OS patients. The networks of these co-occurring genera suggested that dominant bacteria from the nasal and oral cavities closely compete with communicate with each other and might act as a deterrent to pathogenic bacteria in non-OS populations, while dominant pathogens in the nasal and oral cavities of OS patients interact with each other, which might promote the development of disease.

## Discussion

Approximately 10% of CRS cases have an odontogenic aetiology, representing an important subset of patients in whom a response to traditional treatment is not observed [24]. The ecological imbalance hypothesis states that changes in microbial composition associated with disturbance of the local ecological environment are one of the pathogeneses of CRS [25]. Altered nasal microbiota compositions can also be used as biomarkers to predict CRS recurrence [26]. To provide accurate diagnosis and timely treatment of OS, utilization of microbial findings should be performed except for routine assessment of the dental history and status. However, the microbial characterization of maxillary sinusitis associated with odontogenic infection is still poorly understood. Missed diagnosis of OS can lead to more serious infection of the maxillary sinus and subsequent unsatisfactory treatment of dental disease. 16S rRNA sequencing technologies and computational biology have been used to substantially enhance our knowledge of the microbiota associated with CRS and elucidated the pathogenesis of this disease.

From the perspective of bacterial infection, a more diverse community represents an ecosystem with multiple pathogens. Our bacterial analysis results showed that the alpha diversities of the microbiome in both the oral mucosa and nasal secretions of OS patients were higher than those in controls, which might be related to severe odontogenic infection. From the distribution of the dominant bacteria at different taxonomic levels in our study, the microbial structures of oral mucosa from patients and controls were similar, but the microbial structures of nasal secretions from OS patients and controls were wildly inconsistent. This suggests that the microecological imbalance caused by odontogenic infection has a great influence on the nasal microbiome and that treatment programs need to be tailored to nasal microbiome disruption.

The most dominant phylum in OS patients was *Bacteroidota*, while that of the control was *Firmicutes*, either in the oral or nasal cavity. These features highlighted changes in oral and nasal microbiome structure that occurred during odontogenic infection. In terms of dominant genera, the oral microbiome structure of OS patients was similar to that of controls, including *Fusobacterium*, *Porphyromonas* and *Prevotella*. Interestingly, the dominant genera in the nasal microbiome of controls were *Staphylococcus*, *Corynebacterium* and *Cutibacterium*, while those of OS patients were mainly anaerobic bacteria such as *Fusobacterium*, *Porphyromonas* and *Prevotella*. Colonization of these anaerobic bacteria might be due to bacterial translocation caused by odontogenic infection. The presence of anaerobic bacteria in the nasal microbiome of OS patients may indicate potential tissue hypoxia or indicate that the discrete microenvironment within the mucus or bacterial biofilm in OS patients may also be oxygen-limited, allowing anaerobic bacteria to survive [27]. Therefore, antibiotic therapy for OS patients should target anaerobic bacteria.

Moreover, *Porphyromonas* is a common pathogen associated with CRS [28]. *Fusobacterium* infection can also range clinically from local infections to life-threatening infections [29]. In addition, *Treponema*, *Neisseria*, *Capnocytophaga* and *Campylobacter* were abnormally increased in the oral microbiome of OS patients. *Treponema* is proposed to play a key role in the development and progression of periodontal diseases [30]. *Neisseria* is a common bacterium in the oral microbiome, and its composition is known to impact oral microecology, and its counts are closely related to oral health [31]. *Capnocytophaga* is associated with severe periodontitis infection [32]. Additionally, LEfSe analysis showed that the most significantly enriched OTU in both the oral and nasal cavities was *Bacteroidetes*. Increased severity of inflammation in CRS has been shown to be associated with the overall abundance of *Bacteroidetes *[33]. Specific treatment programs for these bacteria should also be emphasized.

We also identified genera that coexisted in the oral and nasal cavities. Network correlation analysis was used to identify interactions and provide a holistic view of microbial ecosystems to better understand the interactions within functional communities. Network diagrams of the co-occurring genera had different core nodes and different connections in control and OS patients. Odontogenic infection affected the network relationship of the oral and nasal microbiomes. In the control group, the bacterial community structure was close, and the genera were interrelated and mutually restricted, forming a relatively stable network system. However, odontogenic infection loosened the relationships among genera, reduced the number of relationships among bacteria, and increased the independence of bacteria. In the bacterial relationship of OS, a network system with *Tannerella* as the core node was formed. As a member of the prototype polybacterial pathogenic consortium of periodontitis [34], *Tannerella* has a promoting effect on the development of OS. A positive relationship between the dominant pathogens in the nasal and oral cavities of OS patients will further promote the development of the disease. This further suggests that therapies targeting the core node bacterium may be more effective in disrupting the complex connections of OS-causing pathogens.

Our research is of unprecedented significance because these findings provide important insights into the microbiological characteristics of the nasal and connected dental regions in OS patients. Therefore, it is important to select the appropriate and targeted therapy for different regions. Our study also has implications for the clinical setting. For example, the identification of bacteria from the oral or nasal cavity during treatment may become useful in routine clinical practice, as corresponding results can help determine the presence of OS as well as antibiotic targeted treatment. Understanding the oral and nasal microbiomes will provide guidance for exploring the function of the OS microbial community and implementing new therapeutic strategies. We suggest that the treatment of OS should consider balanced restoration and strategies for modulating interactions between the dental and nasal microbiome.

## Conclusions

In general, our study has clarified the bacterial characteristics of the sinuses and connected dental regions in OS. Odontogenic infection promotes structural and functional disorders of the nasal microbiome, and the interaction of dominant pathogens in nasal and oral regions may promote the development of OS. Our study provides the microbiological aetiology of the nasal and connected dental regions in OS and is expected to provide novel insights into the diagnosis and therapeutic strategies for OS.

## Supplementary Information

**Additional file 1:** **Figure S1. **Valid sequencing data ：Rarefaction curves of sequencing depth for the collected samples. The number of species detected in each sample (y-axis) increased with the increasing number of sequences per sample (x-axis). All curves showed saturation at approximately 25,000 sequences per sample, indicating that the sequencing depth was adequate to capture all species.

## Data Availability

All the raw sequences have been stored in the NCBI Sequence Read Archive (SRA) with the login number PRJNA930496. The data are available through a link: https://www.ncbi.nlm.nih.gov/sra/?term=PRJNA930496

## Abbreviations

OS: Odontogenic Sinusitis
CRS: Chronic rhinosinusitis
EGNS: Experimental group nasal secretions
CGNS: Control group nasal secretions
EGOM: Experimental group oral mucosa
CGOM: Control group oral mucosa
PCoA: Principal coordinate analysis
OTUs: Operational taxonomic units
LEfSe: Linear discriminant analysis effect size
LDA: Linear discriminant analysis
