# Supplementary material for: Bacterial diversity and community characteristics of the sinus and dental regions in adults with odontogenic sinusitis
Source: BMC Microbiol. 2023 Jul 29;23:201. doi: 10.1186/s12866-023-02917-7 (PMC10386777; doi:10.1186/s12866-023-02917-7)

**Figure S1.**

Valid sequencing data ：Rarefaction curves of sequencing depth for the collected samples. The number of species detected in each sample (y-axis) increased with the increasing number of sequences per sample (x-axis). All curves showed saturation at approximately 25,000 sequences per sample, indicating that the sequencing depth was adequate to capture all species.


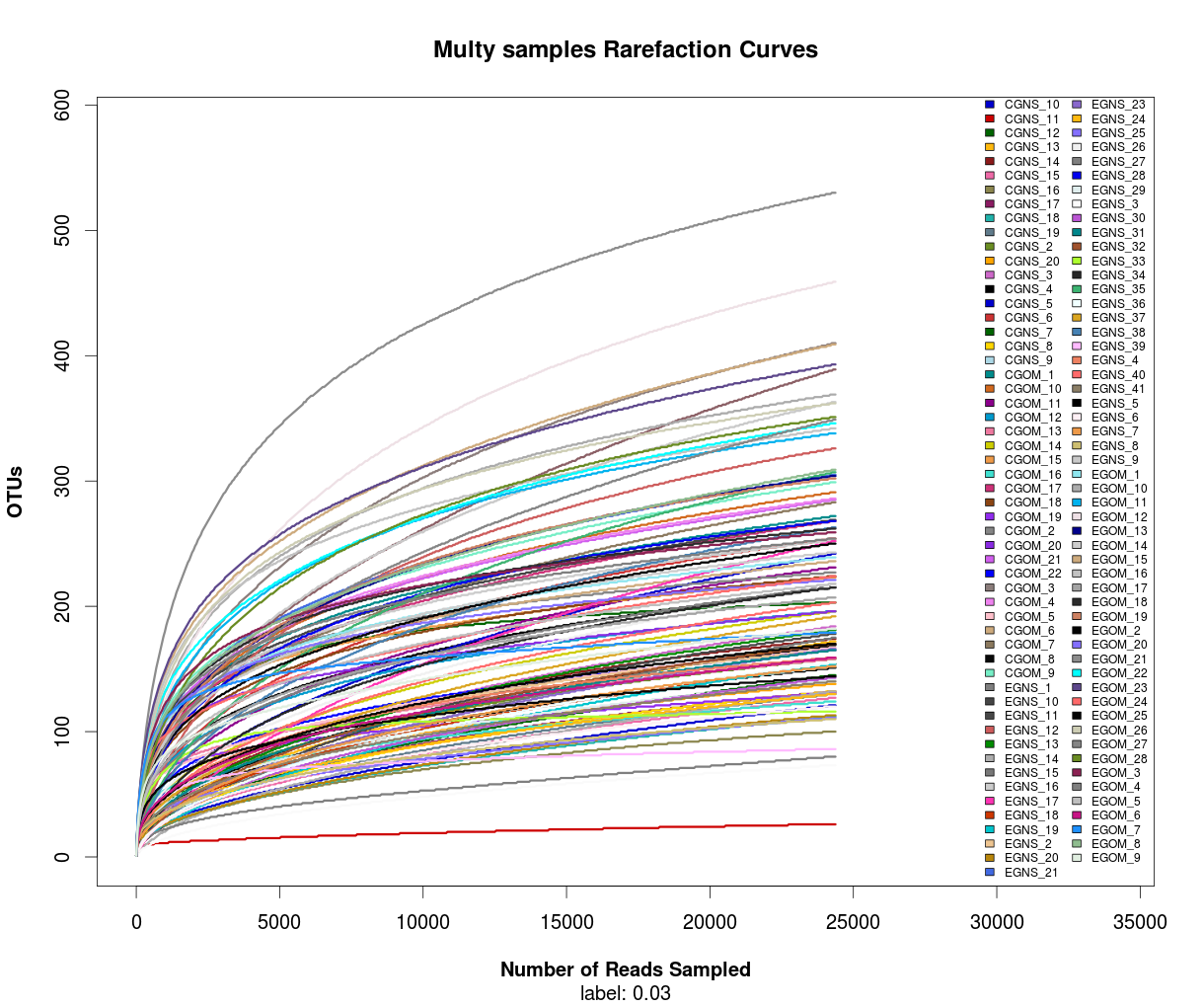

Supplement: Supplementary file 1 — Additional file 1: Figure S1. Valid sequencing data ：Rarefaction curves of sequencing depth for the collected samples. The number of species detected in each sample (y-axis) increased with the increasing number of sequences per sample (x-axis). All curves showed saturation at approximately 25,000 sequences per sample, indicating that the sequencing depth was adequate to capture all species. [file 12866_2023_2917_MOESM1_ESM.docx]
